# Peptide receptor radionuclide therapy alone or in combination with temozolomide plus/minus capecitabine in [^18^F]FDG-positive metastatic neuroendocrine tumors

**DOI:** 10.1007/s00259-025-07606-3

**Published:** 2025-10-21

**Authors:** Gianpaolo di Santo, Giulia Santo, Lukas Wirth, Ariane Kronthaler, Günther Gastl, Angela Djanani, Irene J. Virgolini

**Affiliations:** 1 Department of Nuclear Medicine, Medical University of Innsbruck, Anichstrasse 35, Innsbruck, 6020 Austria; 2 Department of Experimental and Clinical Medicine, “Magna Graecia” University of Catanzaro, Catanzaro, Italy; 3 Department of Internal Medicine V, Medical University of Innsbruck, Innsbruck, Austria; 4 Department of Internal Medicine I, Gastroenterology, Hepatology, Endocrinology & Metabolism, Medical University Innsbruck, Innsbruck, Austria

**Keywords:** Peptide receptor radionuclide therapy, Neuroendocrine tumors, [^18^F]FDG, CAPTEM

## Abstract

**Background:**

Recent data demonstrate that one possibility for increasing Peptide Receptor Radionuclide Therapy (PRRT) results lies in the combination of PRRT with chemotherapy. This study aimed to evaluate response and outcome in [^18^F]FDG-positive metastatic neuroendocrine tumor (mNET) patients treated with PRRT alone or in combination with temozolomide (TEM) plus/minus capecitabine (CAP).

**Methods:**

All mNET patients presented with [^18^F]FDG-positive disease prior to treatment (or retreatment) with PRRT alone or in combination with chemotherapy were retrospectively included in this single-center study. Patients received [^177^Lu]Lu-DOTATATE with an activity of 7.4 GBq alone or combined with TEM (200 mg/kg/5d) plus/minus CAP (1500 mg/kg/14d). Contrast-enhancement CT (ceCT), [^68^Ga]Ga-DOTATOC and [^18^F]FDG PET/CT studies were performed at baseline, after treatment, and every 6 months thereafter. Overall response rate (ORR) and disease control rate (DCR) were calculated for each group. Survival analysis was performed using the Kaplan-Meier method. Adverse events were collected and classified according to the Common Terminology Criteria for Adverse Events (CTCAE) v5.0.

**Results:**

A total of 24 patients were included in the final analysis. Group 1 received PRRT with [^177^Lu]Lu-DOTATATE alone (*n* = 10), group 2 received [^177^Lu]Lu-DOTATATE plus TEM (*n* = 7), and group 3 received [^177^Lu]Lu-DOTATATE plus CAPTEM (*n* = 7). There were no differences between groups in terms of clinicopathological features before treatment. The pancreas was the primary tumor site in 58%, and 92% of patients had more than three liver metastases. Based on [^68^Ga]Ga-DOTATOC PET/CT and ceCT, in group 1 the ORR and DCR were 10% and 50%, respectively. [^18^F]FDG PET/CT showed 2 responders (1 CR, 1 PR). In group 2, the ORR and DCR were 14% and 43%, respectively. [^18^F]FDG PET/CT showed 2 responders (1 CR, 1 PR). In group 3, the ORR and DCR were 71%, respectively. [^18^F]FDG PET/CT showed 5 responders (3 CR, 2 PR). In the latter group, 4/7 patients had not progressed and 5/7 were still alive at the time of analysis after a median follow-up of 31 months.

**Conclusion:**

Our results show PRRT combined with CAPTEM as the most promising regimen, achieving higher response rates in [¹⁸F]FDG-positive mNETs compared with PRRT alone or PRRT plus TEM. Further studies are required to confirm its added value in terms of survival outcomes. No increased toxicity seems to be associated with combination therapy compared to PRRT alone.

**Supplementary Information:**

The online version contains supplementary material available at 10.1007/s00259-025-07606-3.

## Introduction

 Peptide receptor radionuclide therapy (PRRT) currently follows a standardized treatment protocol with [¹⁷⁷Lu]Lu-DOTATATE, administered in four cycles (7.4 GBq each, eight weeks apart), along with amino acid infusion to minimize kidney radiation exposure [1]. The regulatory approval of [¹⁷⁷Lu]Lu-DOTATATE PRRT following the phase 3 NETTER-1 trial [2], together with increased availability through industry, has led to its incorporation into the standard of care. According to ESMO guidelines, PRRT is now recommended for patients with progressive midgut neuroendocrine tumors (NETs) who exhibit homogeneous somatostatin receptor (SSTR) expression across all lesions (G1–G2), as demonstrated by SSTR-based imaging [3]. Results from the NETTER-2 trial may further expand PRRT indications [4]. In addition, although not included in the aforementioned trials, PRRT also represents a valuable treatment option for other metastatic SSTR-expressing tumors [5], such as lung carcinoids or NET of unknown primary [6, 7].

However, limited therapeutic options are available after PRRT in progressive NET patients. A consensus document released in 2021 suggests considering rechallenge PRRT in progressive patients who achieved disease stabilization or remission lasting at least one year after initial PRRT [8]. Notably, data on rechallenge PRRT showed similar efficacy and safety profiles compared to the initial treatment [9, 10].

However, despite the high disease control rate (DCR) reported (i.e., up to 80%), a significant proportion of patients experience only stable disease (SD) or minor response (~ 55%), and only 25% achieve complete (CR) or partial response (PR) following PRRT. Approximately 20% of patients show progressive disease (PD). Overall, PRRT-treated patients exhibit a median progression-free survival (PFS) of 2 years and a median overall survival (OS) of 4 years [11]. In the pre-specified final analysis of OS of the phase 3 NETTER-1 trial [12], with a median follow-up of over 6.3 years, the intention-to-treat population did not reach statistical significance (HR 0.84; 95% CI: 0.60–1.17; *p* = 0.30), likely influenced by a high crossover rate (36%). Specifically, the median OS was 48.0 months in the [¹⁷⁷Lu]Lu-DOTATATE arm versus 36.3 months in the control arm.

In recent years, efforts to improve the outcomes of PRRT have increasingly focused on understanding both intra- and inter-tumoral heterogeneity, along with the ability to tailor personalized treatment strategies for patients with progressive NET [13]. In particular, dual-tracer positron emission tomography/computed tomography (PET/CT) imaging with [⁶⁸Ga]Ga-DOTA-somatostatin analogs (SSA) and [¹⁸F]FDG has proven to be effective in providing a more comprehensive overview of the tumor landscape in NET patients [13, 14]. This approach may help overcome the limitations of single-site biopsy and provide a deeper understanding of disease variability. [^18^F]FDG positivity is widely recognized as a negative prognostic factor, being associated with poorer survival outcomes [15–17]. As tumor grade increases, NETs tend to show reduced SSTR-expression and increased [^18^F]FDG uptake [16, 18]. In addition, the selection of more resistant tumor clones during disease progression and after cancer therapies can enhance tumour aggressiveness [19], possibly resulting in an increased number of FDG-positive lesions. As a result, there is a significant overlap in which some NET patients exhibit high uptake on both [⁶⁸Ga]Ga-DOTA-SSA and [¹⁸F]FDG PET/CT scans [20, 21].

For these patients, a therapeutic approach combining PRRT with chemotherapy may represent an effective strategy for improving treatment results, possibly with an acceptable safety profile.

There are different mechanisms underlying treatment combinations. Specifically, chemotherapeutic agents can halt cells in mitosis — when they are most radiosensitive — by acting as radiation sensitizers [22, 23], or they can be combined at fixed doses to exploit an additive effect [24]. In this scenario, the combination of PRRT with various chemotherapeutic drugs is currently being investigated [25]. In the study by Owen et al. [26], capecitabine (CAP) plus temozolomide (TEM) regimen demonstrated significant anti-tumor activity in both pancreatic (panNET) and non-panNET patients, showing a DCR of 90%, a PFS of 13 months, and an OS of 29.3 months. A Phase 2 study on 33 metastatic NET (mNET) patients treated with PRRT plus CAP (1650 mg/m2/d/14d) revealed 24% PR and 70% SD (i.e., DCR 94%) despite one single case of Grade 3 thrombocytopenia [27]. The same group demonstrated that for on top-treatment with escalating doses of TEM (200 mg/m2/5d), no dose-limiting toxicities were observed [28]. The Australian group [29] reported a PFS of 61.1% for panNETs when treated with PRRT plus CAPTEM, compared to 33.3% for patients treated by CAPTEM only at the 27-month follow-up. However, in their evaluation, no difference was found for mNETs at the 36-month follow-up between both groups. Though, the long-term results have not yet been published [30]. Thus, findings remain controversial and the optimal combination treatment has not yet been established.

In the present study, we aimed to evaluate the treatment response and outcomes of PRRT alone or in combination with chemotherapy (temozolomide plus/minus capecitabine) in [^18^F]FDG-positive patients with advanced metastatic NETs. Additionally, the toxicity profile was collected and reported.

## Methods

### Patients

Patients with advanced, [^18^F]FDG-positive metastatic NET (mNET) were retrospectively identified from our clinical records and included in the study based on the following criteria: (i) inoperable/metastatic, well-differentiated G1–G3 NETs with positive SSTR expression in the majority of known tumor sites, as demonstrated by [^68^Ga]Ga-DOTATOC PET/CT; (ii) at least one positive lesion on [^18^F]FDG PET/CT imaging; and (iii) treatment (or retreatment) with at least two cycles of [^177^Lu]Lu-DOTATATE, either alone or in combination with chemotherapy.

Of note, retreatment with additional PRRT cycles was offered to progressive NET patients (clinical and/or imaging progression), who achieved disease stabilization or remission after initial PRRT.

**Fig. 1:**
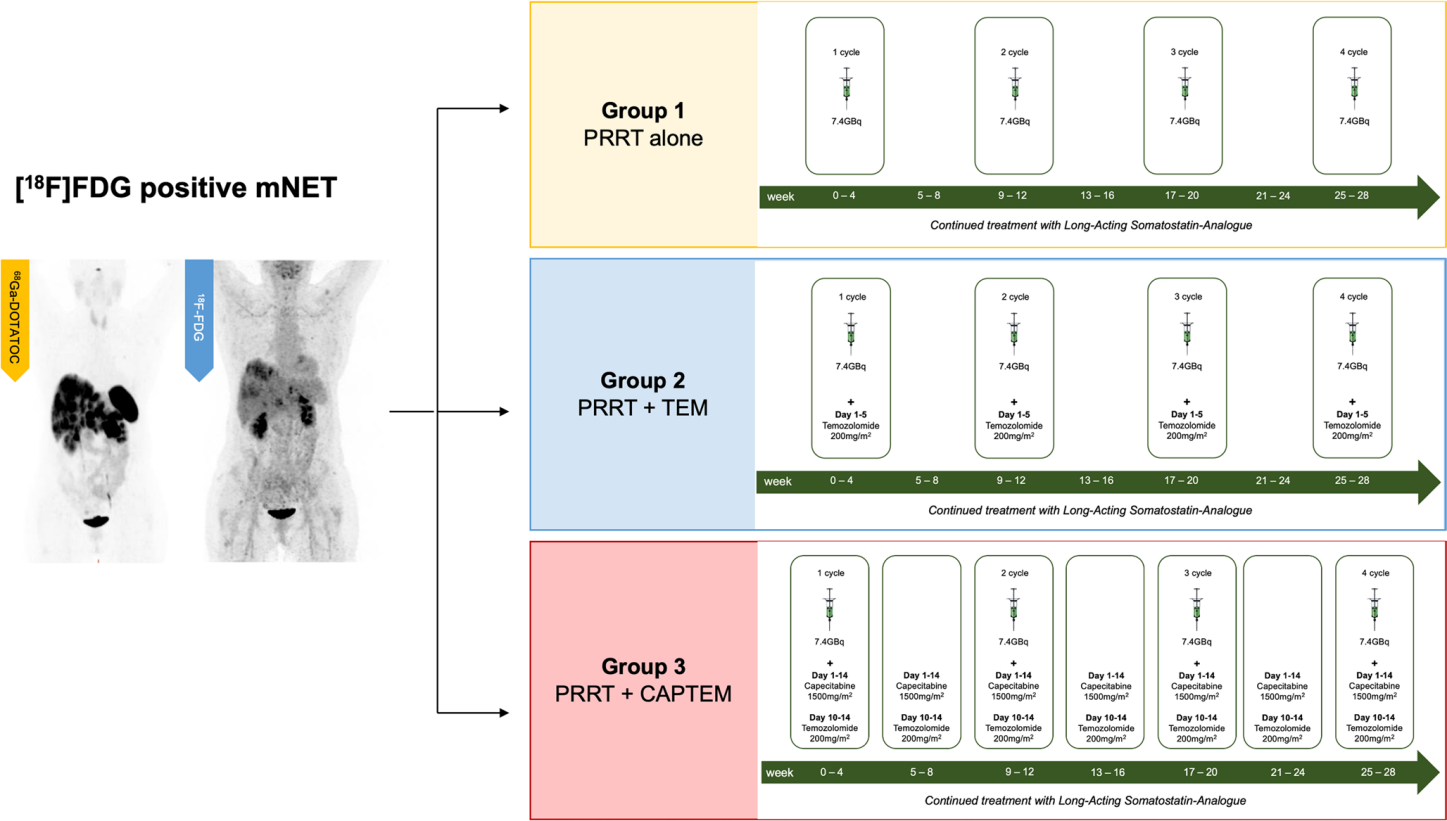
Schematic representation of patient selection method and treatment schemes. All [¹⁸F]FDG-positive metastatic neuroendocrine tumors (mNET) were retrospectively collected. Patients were then divided into three groups: group 1 received PRRT alone, group 2 received PRRT combined with temozolomide (TEM), and group 3 received PRRT combined with capecitabine and temozolomide (CAPTEM)

After inclusion, patients were grouped according to the treatment scheme: all patients received PRRT with an activity of 7.4 GBq of [^177^Lu]Lu-DOTATATE alone or in combination with TEM (200 mg/kg/5d) plus/minus CAP (1500 mg/kg/14d). The administered activity was adjusted in cases of bone marrow, renal or liver function impairment before or during the course of therapy, especially in the elderly. Figure 1 summarizes the patient selection method and the treatment schemes used at the Department of Nuclear Medicine of the Medical University of Innsbruck.

Clinicopathological characteristics of the patients (gender, age, primary tumor site, NET grade, previous local or systemic treatment(s), rechallenge PRRT, site of disease before the start of treatment, administered activity) were retrospectively extracted from medical records.

All procedures performed in this study were in accordance with the principles of the 1964 Declaration of Helsinki and its subsequent amendments [31]. [^177^Lu]Lu-DOTATATE was prepared according to the Austrian Medicinal Products Act AMG § 8 and § 62 [32] and all regulations of the Austrian Agency for Radiation Protection were observed [33]. The study evaluation followed an intention-to-treat approach for all patients, and patients were followed until death.

### Imaging protocol

All scans were performed on a dedicated PET/CT system with time-of-flight capability (Discovery MI; GE Healthcare). A whole-body PET scan, covering the region from the skull vertex to the upper thighs, was acquired in three-dimensional mode 1-hour post-injection with an axial field of view of 20 cm and an emission time of 2 min per bed position. A low-dose CT scan was used for attenuation correction of the PET emission data. All PET images were corrected for random events, scatter, and decay. Image reconstruction was performed using the VUE Point FX iterative reconstruction method (GE Healthcare) without a z-axis filter, in combination with the Q.Clear software package (GE Healthcare), a fully convergent iterative reconstruction method with noise control.

For [⁶⁸Ga]Ga-DOTATOC PET/CT, a standard administered activity of 120 MBq was used. PET Image reconstruction was conducted with Q.Clear (β = 1,000). After administration of a contrast agent (Ultravist-370^®^, Bayer) at a flow rate of 3.5 ml/s and a dose of approx. 1 ml/kg body weight, a diagnostic contrast-enhanced CT (ceCT) scan of the chest, abdomen, and pelvis was performed during expiration.

For [¹⁸F]FDG PET/CT, the administered activity was weight-dependent (2 MBq/kg). Only a low-dose CT was acquired for attenuation correction, and PET image reconstruction was performed using Q.Clear (β = 550).

[⁶⁸Ga]Ga-DOTATOC and [¹⁸F]FDG PET/CTs were performed before treatment, 3 months after the last treatment cycle, and every 6 months thereafter.

### Image review and analysis

All PET/CT images were first visually analyzed using commercially available software (eNTEGRA; GE Healthcare), which allowed for the review of PET, CT, and fused imaging data. At each time-point, [^68^Ga]Ga-DOTATOC and [^18^F]FDG PET images were assessed by two experienced board-certified nuclear medicine physicians. The criteria for a positive finding on PET studies were focal area(s) of increased tracer uptake or diffusely increased uptake, excluding physiological uptake, in comparison with adjacent tissue on axial, coronal, and sagittal images. In addition to visual assessment, [^68^Ga]Ga-DOTATOC and [^18^F]FDG PET/CT scans were analyzed using Affinity 5.0.1 software within the Hermia platform (Hermes Medical Solutions, Stockholm, Sweden). A semi-automatic segmentation tool was applied to delineate the regions of interest (ROIs): the metabolic tumor volume (MTV) and total lesion burden (TLB) from the whole tumor were extracted.

### Response assessment

Response assessment was performed 3 months after the end of treatment and every 6 months thereafter. The metabolic responses observed by [^18^F]FDG PET/CT were classified using PET Response Evaluation Criteria in Solid Tumors (PERCIST). The PERCIST criteria were adapted to evaluate the response in [^68^Ga]Ga-DOTATOC PET/CT. Accordingly, the response findings were classified into 4 groups: complete response (CR), partial response (PR), stable disease (SD), and progressive disease (PD).

RECIST 1.1 was used to determine the morphological tumor response to treatment. The overall response rate (ORR) was defined as CR and PR. DCR was defined as CR + PR + SD.

A lesion-based subanalysis was subsequently performed on corresponding lesions with both [^68^Ga]Ga-DOTATOC and [^18^F]FDG PET/CT uptake. For organs with more than three lesions, only the three with the highest volume were considered.

### Toxicity assessment

Clinical and laboratory tests were carried out before every treatment infusion, at the first restaging, and at every 6-month follow-up visit. Laboratory examinations included parameters for monitoring bone marrow function (i.e., hemoglobin, white blood cell count, and thrombocytes), renal function (i.e., creatinine and glomerular filtration rate), and liver function (i.e., aspartate aminotransferase, alanine aminotransferase, and bilirubin). Adverse events were classified according to the Common Terminology Criteria for Adverse Events (CTCAE) v5.0 [34].

### Statistical analysis

Statistical analysis was performed with IBM SPSS Statistics for Windows version 31.0 (IBM Corp., Armonk, N.Y., USA) and GraphPad Prism version 10.0.0 for Windows (GraphPad Software, Boston, Massachusetts USA). Categorical and continuous variables were analyzed using descriptive statistics. Demographic and individual baseline clinical characteristics were compared using Chi-square test or Fisher’s exact test for categorical variables, while Kruskal Wallis test and one-way ANOVA were used to compare continuous variables according to their distribution. The Shapiro-Wilk test was used to examine the distribution of the data.

Survival analysis was performed using the Kaplan-Meier method. PFS and OS were calculated from the first cycle of treatment. Patients who were alive or progression-free were censored at the date of the last follow-up. Statistical comparison of survival curves was performed using the log-rank test. Probability values (*p*) < 0.05 were considered statistically significant.

All analyses were performed on the whole sample and in the subgroup of patients who underwent rechallenge PRRT.

## Results

### Patients’ characteristics

A total of 24 patients (median age 68 years; 36–80 years) met the inclusion criteria and were included in the analysis. The clinicopathological characteristics of the study cohort are summarized in Table 1. At diagnosis 21/24 patients (87%) had Grade 2 disease, and 14/24 (58%) had a primary panNET. Before treatment initiation, 22/24 patients (92%) had more than three liver metastases, 12/24 (50%) had lymph node metastases and 12/24 (50%) bone involvement. Patients received a median of 4 cycles (range 3–5) of [^177^Lu]Lu-DOTATATE (plus/minus chemo), with a mean cumulative activity of 22.9 GBq (IQR: 19.4–29.9). According to the treatment scheme, 10/24 patients (42%) underwent PRRT alone (group 1), 7 (29%) received PRRT plus TEM (group 2), and 7 (29%) received a combination of PRRT plus CAPTEM (group 3).

**Table 1:** Patients’ characteristics

	PtID	Age	Primary	ki67% diagnosis	Rechallenge setting	SSTR vs. FDG (visual assessment)	No cycles last course	Cumulated activity last course (GBq)
**PRRT alone**	1	74	Sigma	20	YES	SSTR > > FDG	4	16,80
	2	79	Pancreas	10	YES	SSTR = FDG	3	22,40
	3	58	Pancreas	20	NO	SSTR > > FDG	3	23,00
	4	58	Pancreas	8	NO	SSTR > > FDG	4	29,43
	5	64	Pancreas	7	YES	SSTR = FDG	5	30,16
	6	75	Lung	N/A	YES	SSTR > > FDG	4	22,90
	7	68	Pancreas	10	YES	SSTR > FDG	4	16,20
	8	76	Small intestine	N/A	YES	SSTR > > FDG	3	19,10
	9	69	Unknown	N/A	NO	SSTR = FDG	4	21,26
	10	79	Small intestine	20	NO	SSTR > FDG	3	22,00
**PRRT + TEM**	11	78	Pancreas	20	NO	SSTR > > FDG	4	22,50
	12	52	Gastric	8	YES	SSTR > > FDG	5	20,40
	13	72	Pancreas	10	YES	SSTR > > FDG	2	14,80
	14	36	Unknown	20	YES	SSTR > > FDG	4	30,70
	15	75	Pancreas	10	YES	SSTR > > FDG	4	30,22
	16	55	Lung	70	NO	SSTR > FDG	4	30,00
	17	42	Pancreas	30	YES	SSTR = FDG	4	17,50
**PRRT + CAPTEM**	18	60	Gastric	11	NO	SSTR > FDG	4	30,00
	19	59	Pancreas	14	NO	SSTR > > FDG	4	28,70
	20	51	Pancreas	10	YES	SSTR = FDG	4	29,50
	21	68	Pancreas	15	NO	SSTR = FDG	4	29,54
	22	63	Sigma	16	YES	SSTR > FDG	4	23,00
	23	68	Pancreas	8	YES	SSTR > > FDG	4	30,32
	24	80	Pancreas	20	YES	SSTR = FDG	3	18,86

*CAP* capecitabine, *FDG *[^18^F]Fluorodeoxyglucose, *N/A* not available, *PRRT* peptide receptor radionuclide therapy, *SSTR* somatostatin receptor, *TEM* temozolomide

None of the baseline clinicopathological variables analyzed including age, gender, primary tumor site, NET grade, previous local or systemic treatment(s), presence of bone or lymph node metastases, rechallenge PRRT, and cumulative activity, showed significant differences among treatment groups (Supplementary Table 1). In addition, considering the whole tumor burden before treatment, measured on both [^68^Ga]Ga-DOTATOC and [^18^F]FDG PET/CT, there were no significant differences between the three treatment groups (Supplementary Table 2).

Fifteen patients (63%) had previously undergone treatment with [^90^Y]Y-DOTATOC/[^177^Lu]Lu-DOTATATE (rechallenge setting), receiving a mean cumulative administered activity at initial PRRT of 40.1 GBq (IQR: 29.5–46.1). Median time between the last cycle of the initial PRRT and the first cycle of rechallenge was 26 months (range: 11–43 months). When considering the rechallenge subgroup, clinicopathological variables and total tumor burden did not differ among the treatment groups (data not shown).

### Response assessment

#### Patient-based analysis (n = 24)

According to CT-based and [^68^Ga]Ga-DOTATOC PET/CT, the response distribution among the whole sample was as follows: 1 patient (4%) achieved CR, 6 patients (25%) had PR, 6 patients (25%) had SD, and 11 patients (46%) had PD.

Group 1 (PRRT alone): CT-based and [^68^Ga]Ga-DOTATOC PET/CT evaluation showed 1 CR, 4 SD, and 5 PD with an ORR and DCR of 10% and 50%, respectively. [^18^F]FDG PET/CT showed 2 responders (1 CR, 1 PR) and 7 non-responders (1 SD, 6 PD). In one patient with SD on other imaging modalities, post-treatment [^18^F]FDG PET/CT restaging was not available.

Group 2 (PRRT + TEM): CT-based and [^68^Ga]Ga-DOTATOC PET/CT evaluation showed 1 PR, 2 SD, and 4 PD. The ORR and DCR were 14% and 43%, respectively. [^18^F]FDG PET/CT showed 2 responders (1 CR, 1 PR) and 5 non-responders (1 SD, 4 PD).

Group 3 (PRRT + CAPTEM): CT-based and [^68^Ga]Ga-DOTATOC PET/CT evaluation showed 5 PR and 2 PD. The ORR and DCR were 71%. [^18^F]FDG PET/CT showed 5 responders (3 CR, 2 PR) and 2 patients with PD. The ORR was statistically associated with treatment scheme (10% vs. 14% vs. 71%, *p* = 0.01).

Table 2 shows response to treatment according to different imaging modalities (ceCT, [^68^Ga]Ga-DOTATOC, and [^18^F]FDG PET/CT) across different treatment groups.

**Table 2:** Patient-based response to treatment according to different imaging modalities (ceCT, [^68^Ga]Ga-DOTATOC, and [^18^F]FDG PET/CT) across different groups

	PRRT alone			PRRT + TEM			PRRT + CAPTEM		
	ceCT	[^68^Ga]Ga-DOTATOC	[^18^F]FDG	ceCT	[^68^Ga]Ga-DOTATOC	[^18^F]FDG	ceCT	[^68^Ga]Ga-DOTATOC	[^18^F]FDG
CR	1	1	1	0	0	1	0	0	3
PR	0	0	1	1	1	1	5	5	2
SD	4	4	1	2	2	1	0	0	0
PD	5	5	6	4	4	4	2	2	2

*CAP* capecitabine, *ceCT* contrast-enhanced computed tomography, *CR* complete response, *PD* progressive disease, *PR* partial response, *PRRT* peptide receptor radionuclide therapy, *SD* stable disease, *TEM* temozolomide

**Fig. 2:**
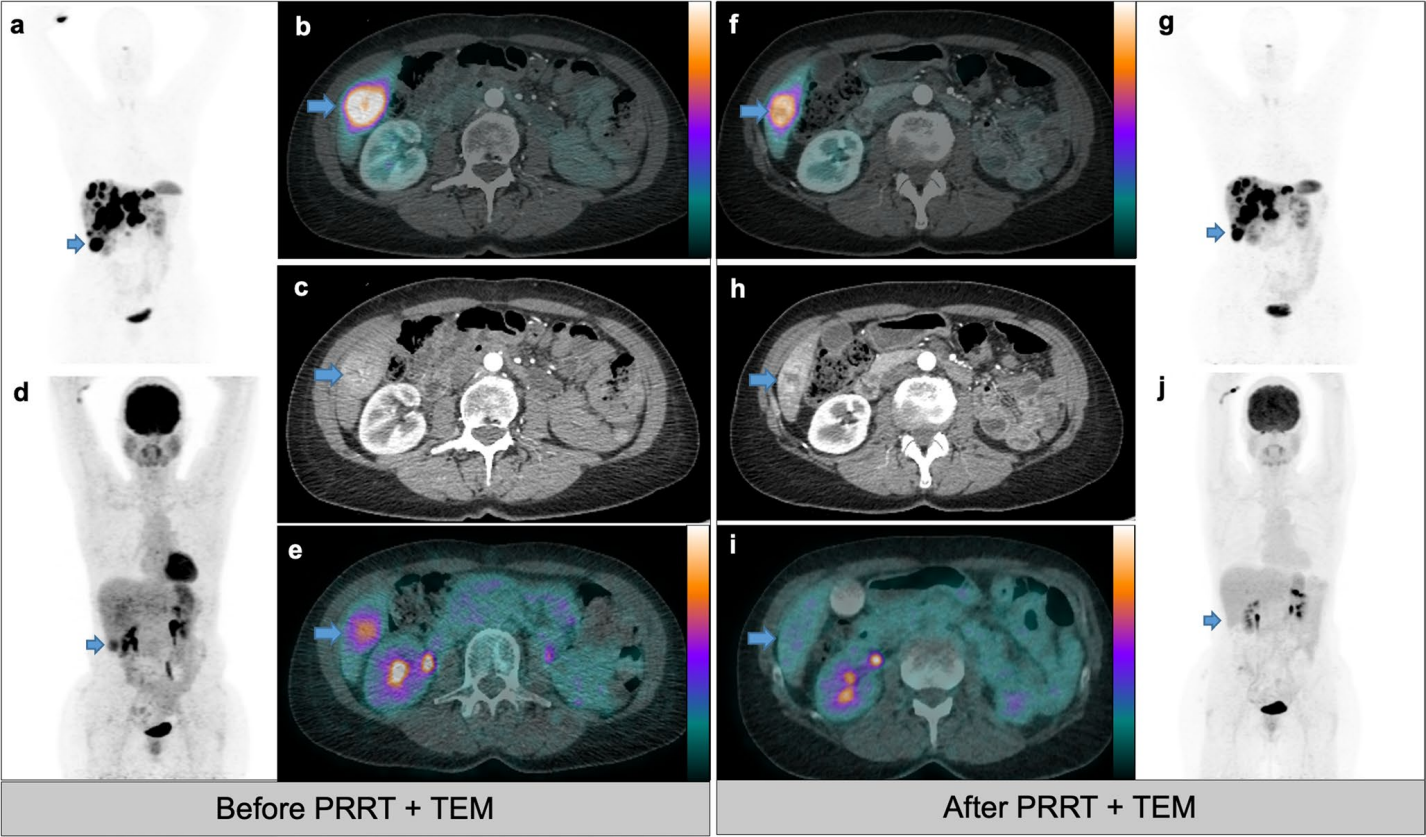
Patient treated with PRRT plus temozolomide (ID12). A 52-year-old female patient diagnosed in 2008 with gastric NET (G2, Ki67 8%). After progression under cold somatostatin analogs and previous PRRT, she presented diffuse liver metastases (a-e). The patient underwent a combination therapy of (rechallenge) peptide receptor radionuclide therapy (PRRT) plus temozolomide (TEM) 200 mg/m^2^/5d. Imaging performed after the end-of-treatment demonstrated stable disease on both [⁶⁸Ga]Ga -DOTATOC PET/CT (f,g) and ceCT (h) scans. Notably, the FDG-positive liver lesion in segment VI (d,e) exhibited a complete response after treatment (i,j), though a minor response was observed on [⁶⁸Ga]Ga-DOTATOC PET/CT and CT (blue arrows). The patient had a progression-free survival (PFS) of 25 months and an overall survival (OS) of 29 months

**Fig. 3:**
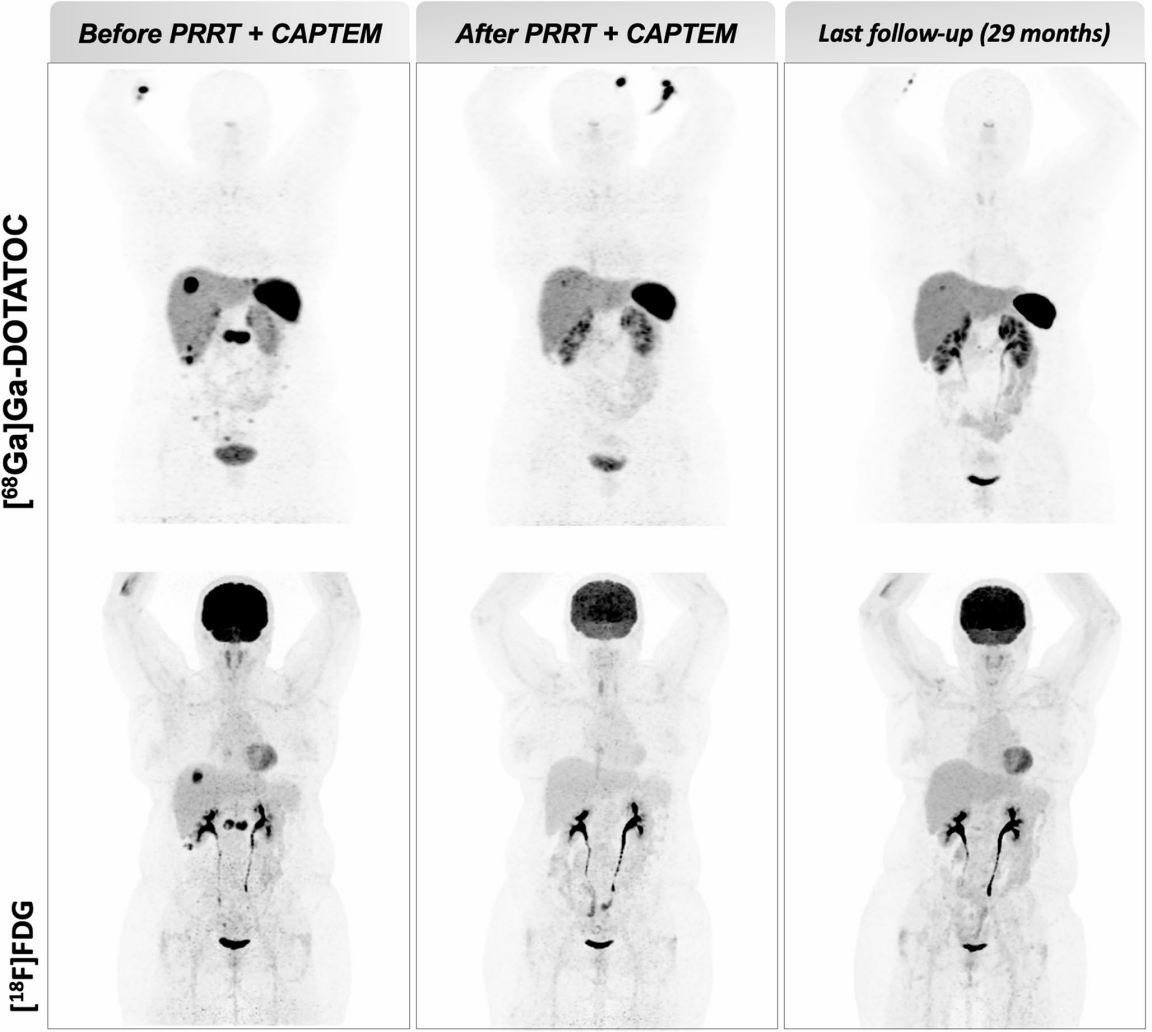
Patient treated with PRRT plus capecitabine and temozolomide (ID18). A 60-year-old female patient diagnosed in 2018 with gastric NET (G2, Ki67 11%) progressed on cold somatostatin analogues and presented with locoregional recurrence, as well as diffuse lymph node, liver, and peritoneal metastases. She underwent combination therapy with peptide receptor radionuclide therapy (PRRT), capecitabine, and temozolomide (CAPTEM). Imaging performed after the end-of-treatment showed a partial response on [⁶⁸Ga]Ga-DOTATOC PET/CT, while [¹⁸F]FDG imaging demonstrated a complete response to treatment. The patient remains alive and progression-free after 29 months of follow-up

Figure 2 and Figure 3 describe two clinical cases extracted from group 2 and 3, respectively.

#### Lesion-based analysis

At pre-treatment assessment, in 23 out of 24 patients, all [^18^F]FDG-positive lesions were also positive on [^68^Ga]Ga-DOTATOC imaging. Only one patient had lung lesions that were [^18^F]FDG-positive but [^68^Ga]Ga-DOTATOC-negative (ID16, belonging to group 2).

A total of 64 corresponding lesions were analyzed: 39 liver lesions, 6 lymph node lesions, 11 bone lesions, 4 lung lesions, and 4 abdominal masses. The response of lesions showing uptake of both tracers was compared across groups. Table 3 reports the distribution of lesions among groups and the corresponding response on [^68^Ga]Ga-DOTATOC and [^18^F]FDG PET/CT, respectively. The distribution of treatment responses across the three groups differed significantly for both [^68^Ga]Ga-DOTATOC PET/CT (*p* = 0.0002) and [^18^F]FDG PET/CT (*p* = 0.0067). Specifically, group 3 showed a higher proportion of CR or PR compared to groups 1 and 2, while group 1 had the highest proportion of PD. Supplementary Table 3 presents lesion responses according to disease site across groups.

**Table 3:** Corresponding lesion-based results of treatment responses among groups

Total lesions *n* = 64	[^68^Ga]Ga-DOTATOC				[^18^F]FDG			
	CR	PR	SD	PD	CR	PR	SD	PD
PRRT alone (*n* = 19)	1	3	2	13	7	2	2	8
PRRT + TEM (*n* = 20)	1	5	8	6	5	6	1	8
PRRT + CAPTEM (*n* = 25)	7	7	10	1	17	5	2	1

Legend: *CAP* capecitabine *CR* complete response, *PR* partial response, *PRRT* peptide receptor radionuclide therapy, *SD* stable disease, *TEM * temozolomide, *PD * progressive disease

#### Rechallenge subgroup

On per patient-based analysis (*n* = 15), according to CT-based and [^68^Ga]Ga-DOTATOC PET/CT the ORR was 0%, 20% and 50% in group 1, 2 and 3, respectively and DCR was 50% vs. 40% vs. 50%, respectively. On [^18^F]FDG PET/CT the ORR was 20%, 40%, and 50% in group 1, 2 and 3, respectively, and DCR was 40% vs. 40% vs. 50%, respectively (Supplementary Table 4).

On per lesion-based analysis, a total of 32 corresponding lesions were analyzed. Group 3 showed a higher proportion of CR or PR compared to groups 1 and 2, on both [^68^Ga]Ga-DOTATOC and [^18^F]FDG PET/CT. However, the difference between groups were not significant (Supplementary Fig. 1).

### Survival analysis

After a median follow-up of 24 months (range 7–83 months), 16/24 patients experienced disease progression, and 15/22 patients had died. Two patients who showed progression at the first restaging were lost at follow-up.

**Fig. 4:**
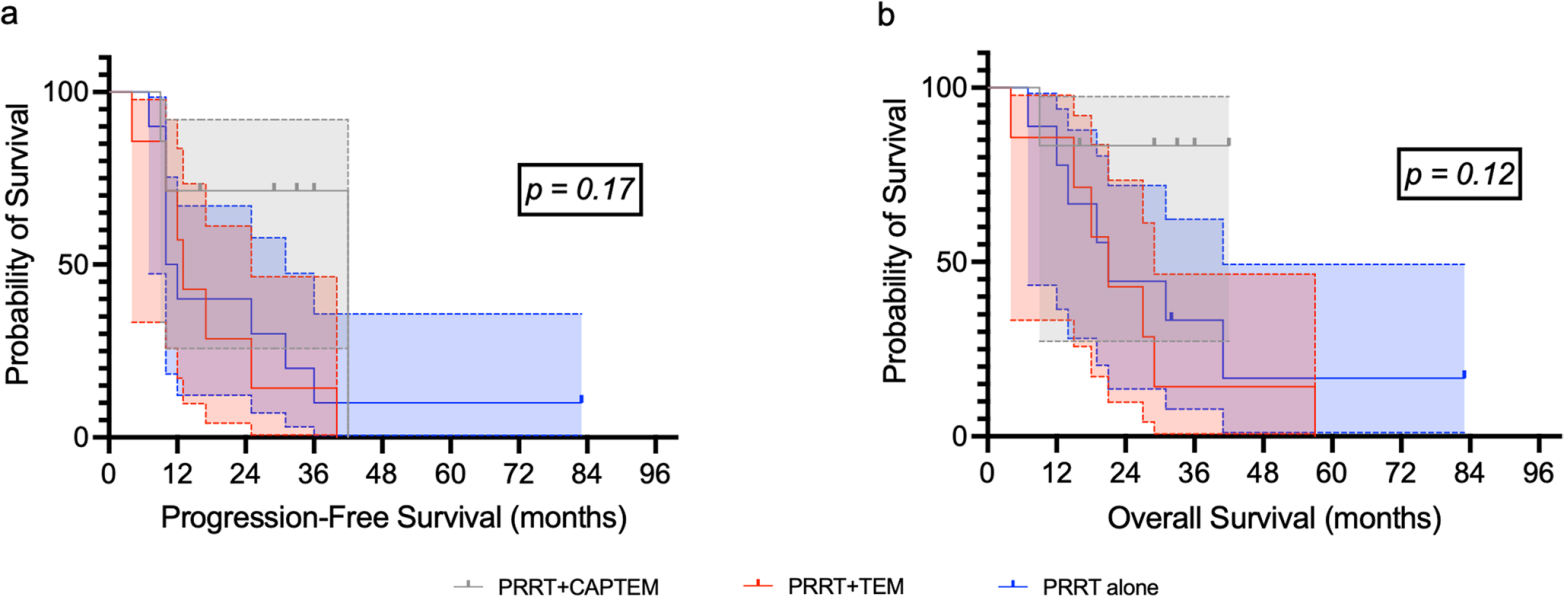
Kaplan-Meir curves for PFS (**a**) and OS (**b**) of different treatment group

The mPFS was 10 months (95% CI, 1.5–24.5 months) for group 1, 13 months (95% CI, 6.9–13.1 months) for group 2, and 42 months (95% CI: NA) for group 3. The mOS was 21 months (95% CI: 16.0–38.0 months) for group 1, 21 months (95% CI: 15.2–26.8 months) for group 2, and was not reached for group 3. Notably, in group 3, 4/7 patients had not progressed, and 5/7 were still alive at the time of analysis (median follow-up 31 months, range: 9–42 months). No significant differences were found in either PFS (*p* = 0.17) or OS (*p* = 0.12) between the groups (Fig. 4). When considering rechallenge subgroup alone, mPFS and mOS were not differed between treatment groups (data not shown).

### Safety profile

Follow-up data were available for 20 of the 24 patients, as 4 died during or immediately after the treatment period. The median follow-up time from the first cycle of PRRT (± chemotherapy) was 25.5 months (range: 4–78 months). Two patients (ID1 and ID13) were lost at follow-up at 4 and 9 months, respectively. The remaining 18 patients were monitored after restaging for a median of 14 months (range: 3–74 months).

Overall, 14/20 patients experienced hematological and/or renal toxicities of any grade, while high-grade (CTCAE > 2) hematological toxicities occurred in 5 of 20 (25%) patients, two of them also developed grade 3 renal impairment.

In group 1 (PRRT alone), one patient (ID4) developed transient grade 3 anemia, grade 4 thrombocytopenia, and grade 3 renal impairment four months after the last treatment cycle. Another patient (ID8) showed only transient G4 anemia and G3 thrombocytopenia one month after the end of treatment.

In group 2 (PRRT + TEM), two patients experienced grade 3/4 toxicities: patient ID12 developed grade 3 anemia, grade 4 thrombocytopenia, and grade 3 renal impairment two to three months after the last treatment cycle; patient ID15 experienced transient grade 3 anemia and grade 3 thrombocytopenia during the last cycle. Grade 3 transient anemia was also registered in one patient (ID16) seven months after the end of treatment.

In group 3 (PRRT + CAPTEM), no grade 3/4 hematological or renal toxicities were observed.

All-grade toxicities across the treatment groups are summarized in Table 4. No significant differences were observed among groups in the occurrence of any-grade adverse events (*p* = 0.23) or high-grade toxicities (*p* = 0.69). Similarly, the incidence of both any-grade (*p* = 0.66) and high-grade toxicities (*p* = 0.66) did not differ between patients treated in the rechallenge versus non-rechallenge setting.

**Table 4:** Toxicity profiles by treatment group from the start of treatment

	PtID	Rechallenge setting	Hematological toxicities	Kidney toxicity
**PRRT alone**	ID1	YES	G2 lymphocytopenia	--
	ID2	YES	--	G2
	ID4	NO	G3 anemia (transient) G4 thrombocytopenia	G3
	ID7	YES	G2 lymphocytopenia	G2
	ID8	YES	G4 anemia (transient) G3 lymphocytopenia (transient)	G2
**PRRT + TEM**	ID11	NO	G2 anemia G2 lymphocytopenia	G2 (transient)
	ID12	YES	G3 anemia G4 thrombocytopenia	G3
	ID13	YES	G2 anemia G2 lymphocytopenia	G2
	ID14	YES	--	G2
	ID15	YES	G3 anemia G3 thrombocytopenia	G2 (transient)
	ID16	NO	G3 anemia (transient)	--
**PRRT + CAPTEM**	ID18	NO	G1 leukopenia (transient) G1 anemia (transient) G1 thrombocytopenia (transient)	G2
	ID19	NO	G1 anemia	--
	ID23	YES	G1 anemia	G1 (transient)

*PRRT *Peptide receptor radionuclide therapy, *CAP* capecitabine, *TEM* temozolomide

## Discussion

The concept of combining different treatment modalities in cancer patients has gained increasing attention in clinical research. In this context, the potential combination of chemotherapeutic agents with PRRT in mNET patients is currently under investigation.

Neuroendocrine tumors are characterized by considerable inter- and intra-lesional heterogeneity [13]. This heterogeneity can be explored using dual-tracer [^68^Ga]Ga-DOTA-SSA/[¹⁸F]FDG PET/CT imaging, which helps to identify patients with more aggressive disease who may benefit from combination therapy. In this study, we reported our experience with [¹⁸F]FDG-positive mNET patients treated with different therapeutic regimens, aiming to evaluate potential differences among treatment approaches. It is well established that more aggressive phenotypes (e.g., higher Ki-67 index) exhibit increased [¹⁸F]FDG uptake, and that [¹⁸F]FDG positivity is highly prognostic across all NET grades [35]. Furthermore, previous studies have shown that a subset of low-grade NETs may also present with [¹⁸F]FDG-positive disease, which requires appropriate management [16]. Our data revealed a high proportion (65%) of patients undergoing PRRT rechallenge with positive [¹⁸F]FDG scans. Following selective pressure from previous therapies such as PRRT, more aggressive tumor clones may drive disease progression, potentially reducing the efficacy of PRRT monotherapy [36]. Several ongoing clinical trials are currently exploring treatment regimens that combine PRRT with chemotherapy, including CAP, TEM, and 5-fluorouracil (5-FU) [37]. Multi-agent chemotherapy in combination with PRRT is thus emerging as a potentially optimized therapeutic approach [38, 39]. However, to the best of our knowledge, no prior studies have directly compared different combination regimens against PRRT alone. In our preliminary analysis, patients treated with PRRT plus CAPTEM achieved the highest ORR (71%), compared with PRRT plus TEM (14%) and PRRT alone (10%). Lesion-based analyses further confirmed higher response rates in the PRRT plus CAPTEM group compared to the other treatment regimens. Yordanova et al. [40] reported a DCR of 55% in 15 NET patients treated with TEM alone (*n* = 3) or CAPTEM (*n* = 12), based on CT evaluation. When assessed with [¹⁸F]FDG and [^68^Ga]Ga-DOTATOC PET/CT, DCRs were 28% and 44%, respectively. In our study, a higher DCR (71%) was observed. These findings are consistent with those of Parghane et al., who reported a DCR of 84% in 38 NET patients treated with “sandwich CAPTEM-PRRT” [41]. Importantly, even in the rechallenge subgroup, the trend of higher responses was maintained in the PRRT plus CAPTEM group compared with the other groups. This suggests that an alternative therapeutic strategy such as CAPTEM-PRRT may provide a valuable option for improving outcomes, particularly in patients with more aggressive disease biology who progress after initial PRRT.

With respect to survival outcomes, no statistically significant differences were observed among the three treatment groups in terms of PFS or OS, which was expected given the limited sample size. Nonetheless, it is noteworthy that in patients treated with PRRT plus CAPTEM, the median PFS was 42 months (+ 29 months compared to PRRT plus TEM; +31 months compared to PRRT alone), while the median OS was not reached after a median follow-up of 31 months. At the time of analysis, 4/7 patients had not progressed and 5/7 were still alive. Interestingly, survival curves did not differ between patients treated with PRRT alone and those treated with PRRT plus TEM.

Regarding safety profile, our findings are consistent with previously published data [40–43], with the majority of patients experiencing low-grade and transient adverse events. Notably, no high-grade toxicities were observed in the PRRT plus CAPTEM group during the follow-up period. Two cases of grade 3 renal failure occurred: one in a patient treated with PRRT alone and one in a patient treated with PRRT plus TEM. However, only the latter had previously received [^90^Y]Y-DOTATOC. Nonetheless, no significant difference in adverse event rates was detected between rechallenge and non-rechallenge settings. Overall, combination therapy did not appear to increase toxicity compared with PRRT alone.

This study has several limitations that should be acknowledged. In addition to its retrospective design and small sample size, the inclusion of patients who had undergone various prior treatment lines may have influenced the results. Moreover, differences in radiopharmaceuticals used during the first PRRT course ([^90^Y]Y-DOTATOC and/or [^177^Lu]Lu-DOTATATE), as well as variations in administered activity and treatment cycles, may represent potential confounders. Finally, the relatively short follow-up after treatment (i.e., median 14 months) may have limited the possibility to assess long-term toxicities such as myelodysplastic syndrome (MDS) or acute myeloid leukemia (AML).

## Conclusion

Although prospective data are still needed, the combination of PRRT and chemotherapy is emerging as an effective treatment option for patients with progressive mNETs. Dual-tracer imaging with [⁶⁸Ga]Ga-DOTATOC and [¹⁸F]FDG PET/CT may help guide the selection of patients who are most likely to benefit from combined treatment. Our results show PRRT combined with CAPTEM as the most promising regimen, achieving higher response rates in [¹⁸F]FDG-positive mNETs compared with PRRT alone or PRRT plus TEM. However, further studies are required to confirm its added value in terms of survival outcomes. No increased toxicity seems to be associated with combination therapy compared to PRRT alone.

## Supplementary Information

Below is the link to the electronic supplementary material.

ESM 1 (DOCX 121 KB)

## Data Availability

The datasets generated during and/or analyzed during the current study are available from the corresponding author upon reasonable request.
