# Supplementary material for: Peptide receptor radionuclide therapy alone or in combination with temozolomide plus/minus capecitabine in [18F]FDG-positive metastatic neuroendocrine tumors
Source: Eur J Nucl Med Mol Imaging. 2025 Oct 21;53(3):1927–38. doi: 10.1007/s00259-025-07606-3 (PMC12860813; doi:10.1007/s00259-025-07606-3)
Supplement: Supplementary file 1 — (DOCX 121 KB) [file 259_2025_7606_MOESM1_ESM.docx]

**Peptide Receptor Radionuclide Therapy alone or in combination with Temozolomide plus/minus Capecitabine in [^18^F]FDG-positive Metastatic Neuroendocrine Tumors**

Gianpaolo di Santo^1^, Giulia Santo^1,2^, Lukas Wirth^1^, Ariane Kronthaler^1^, Günther Gastl^3^, Angela Djanani^4^, Irene J. Virgolini^1^

^1^ Department of Nuclear Medicine, Medical University of Innsbruck, Innsbruck, Austria.

^2^ Department of Experimental and Clinical Medicine, "Magna Graecia" University of Catanzaro, Catanzaro, Italy.

^3^ Department of Internal Medicine V, Medical University of Innsbruck, Innsbruck, Austria.

^4^ Department of Internal Medicine I, Gastroenterology, Hepatology, Endocrinology & Metabolism, Medical University Innsbruck, Innsbruck, Austria.

**Corresponding author:**

Irene J. Virgolini

Department of Nuclear Medicine, Medical University of Innsbruck, Anichstrasse 35, 6020

Innsbruck, Austria

Tel: +43-512-504-22651, fax: +43-512-504-22659

e-mail: [irene.virgolini@i-med.ac.at](mailto:irene.virgolini@i-med.ac.at)

|  | **PRRT alone** | | **PRRT + TEM** | | **PRRT + CAPTEM** | | ***p*** |
| --- | --- | --- | --- | --- | --- | --- | --- |
|  | n | % of the available | n | % of the available | n | % of the available |  |
| **Total** | 10 |  | 7 |  | 7 |  |  |
| **Age (IQR)** | 71.5 (62.5 – 76.75) | | 55 (42 – 75) | | 63 (59 - 68) | | 0.24 |
| **Gender**  Male  Female | 8  2 | 80  20 | 6  1 | 86  14 | 4  3 | 57  43 | 0.42 |
| **Primary tumor site**  Pancreas  Midgut  Gastric  Lung  Unknown origin | 5  3  0  1  1 | 50  30  10  10 | 4  0  1  1  1 | 58  14  14  14 | 5  1  1  0  0 | 72  14  14 | 0.66 |
| **NET Grade**  2  3 | 6  1 | 86  14 | 6  1 | 86  14 | 7  0 | 100 | 0.58 |
| **Prior local therapy for metastatic disease** | 3 | 43 | 2 | 29 | 2 | 29 | 0.80 |
| **Prior systemic treatment other than SSA** | 5 | 71 | 3 | 43 | 1 | 14 | 0.10 |
| **Bone metastases** | 7 | 70 | 2 | 29 | 3 | 43 | 0.22 |
| **Lymph node metastases** | 4 | 40 | 5 | 72 | 3 | 43 | 0.40 |
| **Rechallenge** | 6 | 60 | 5 | 72 | 4 | 57 | 0.84 |
| **Cumulated activity (IQR)**  **(including rechallenge)** | 38.3 (22.7 – 43.3) | | 57.2 (30.0 – 61.3) | | 41.5 (29.5 – 59.8) | | 0.31 |
| **Cumulated activity last course (IQR)** | 22.1 (18.5 – 24.5) | | 22.5 (17.5 – 30.2) | | 29.5 (23.0 – 30.0) | | 0.23 |

**Supplementary Table 1.** Clinicopathological characteristics among treatment groups

|  | PRRT alone | PRRT+TEM | PRRT+CAPTEM | *p* |
| --- | --- | --- | --- | --- |
| SSA_MTV (±SD) | 323.5 ± 405.1 | 281.2 ± 259.0 | 281.1 ± 268.6 | 0.96 |
| SSA_TLB (±SD) | 4706.38 ±7515.4 | 5765.86 ±8177.0 | 3959.3±3701.4 | 0.88 |
| FDG_MTV (±SD) | 82.7± 109.7 | 67.8± 56.9 | 126.2 ±123.1 | 0.54 |
| FDG_TLB (±SD) | 333.9 ±414.3 | 310.14 ±272.2 | 974.7 ±1129.5 | 0.15 |

**Supplementary Table 2.** Whole tumor burden measured by metabolic tumor volume (MTV) and total lesion burden (TLB) before treatment among treatment groups

**Supplementary Table 3.** Lesion responses according to disease site across treatment groups.

| **Group 1 (n=19)** | [^68^Ga]Ga-DOTATOC | | | | [^18^F]FDG | | | |
| --- | --- | --- | --- | --- | --- | --- | --- | --- |
|  | CR | PR | SD | PD | CR | PR | SD | PD |
| Liver (=11) | 1 |  |  | 10 | 7 |  |  | 4 |
| Bone (n=6) |  | 2 | 1 | 3 |  |  | 2 | 4 |
| Lymph node (n=1) |  |  | 1 |  |  | 1 |  |  |
| Abdominal mass (n=1) |  | 1 |  |  |  | 1 |  |  |
| **Group 2 (n=20)** | [^68^Ga]Ga-DOTATOC | | | | [^18^F]FDG | | | |
|  | CR | PR | SD | PD | CR | PR | SD | PD |
| Liver (n=12) |  | 3 | 3 | 6 | 4 | 2 | 1 | 5 |
| Bone (n=3) |  |  | 3 |  |  | 3 |  |  |
| Lymph node (n=1) |  |  | 1 |  |  |  |  | 1 |
| Abdominal mass (n=1) |  | 1 |  |  |  | 1 |  |  |
| Lung (n=3) | 1 | 1 | 1 |  | 1 |  |  | 2 |
| **Group 3 (n=25)** | [^68^Ga]Ga-DOTATOC | | | | [^18^F]FDG | | | |
|  | CR | PR | SD | PD | CR | PR | SD | PD |
| Liver (n=16) | 3 | 4 | 9 |  | 10 | 5 | 1 |  |
| Bone (n=2) |  |  | 1 | 1 |  |  | 1 | 1 |
| Lymph node (n=4) | 2 | 2 |  |  | 4 |  |  |  |
| Abdominal mass (n=2) | 1 | 1 |  |  | 2 |  |  |  |
| Lung (n=1) | 1 |  |  |  | 1 |  |  |  |


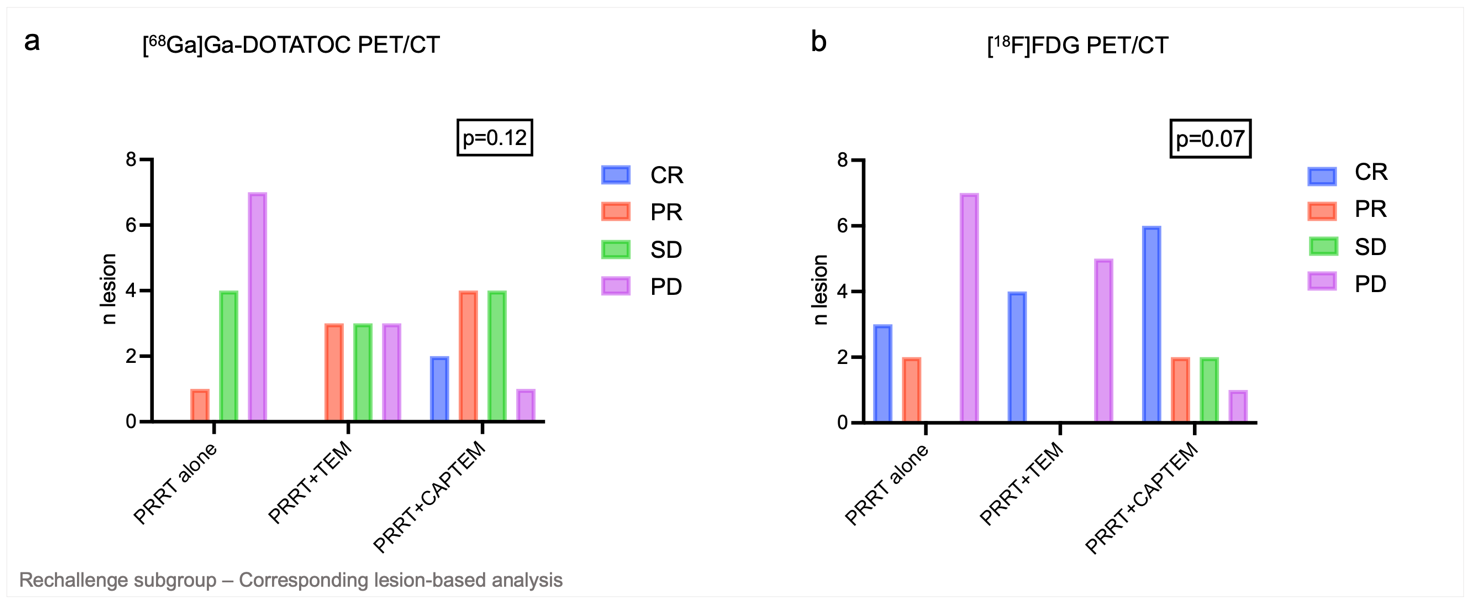


**Supplementary Fig. 1** Corresponding lesion-based analysis in rechallenge subgroup based on a) [^68^Ga]Ga-DOTATOC and b) [^18^F]FDG PET/CT response evaluation.
